# Geomagnetic secular variation forecast using the NASA GEMS ensemble Kalman filter: A candidate SV model for IGRF-13

**DOI:** 10.1186/s40623-020-01324-w

**Published:** 2021-02-11

**Authors:** Andrew Tangborn, Weijia Kuang, Terence J. Sabaka, Ce Yi

**Affiliations:** 1 Joint Center for Earth Systems Technology, University of Maryland Baltimore County, Baltimore, MD USA; 2 Geodesy and Geophysics Laboratory, Goddard Space Flight Center, Greenbelt, MD USA

**Keywords:** Geomagnetic Secular Variation, Data Assimilation, Geodynamo Model

## Abstract

**Abstract:**

We have produced a 5-year mean secular variation (SV) of the geomagnetic field for the period 2020–2025. We use the NASA Geomagnetic Ensemble Modeling System (GEMS), which consists of the NASA Goddard geodynamo model and ensemble Kalman filter (EnKF) with 400 ensemble members. Geomagnetic field models are used as observations for the assimilation, including *gufm1* (1590–1960), CM4 (1961–2000) and CM6 (2001–2019). The forecast involves a bias correction scheme that assumes that the model bias changes on timescales much longer than the forecast period, so that they can be removed by successive forecast series. The algorithm was validated on the time period 2010-2015 by comparing with CM6 before being applied to the 2020–2025 time period. This forecast has been submitted as a candidate predictive model of IGRF-13 for the period 2020–2025.

**Graphical abstract:**

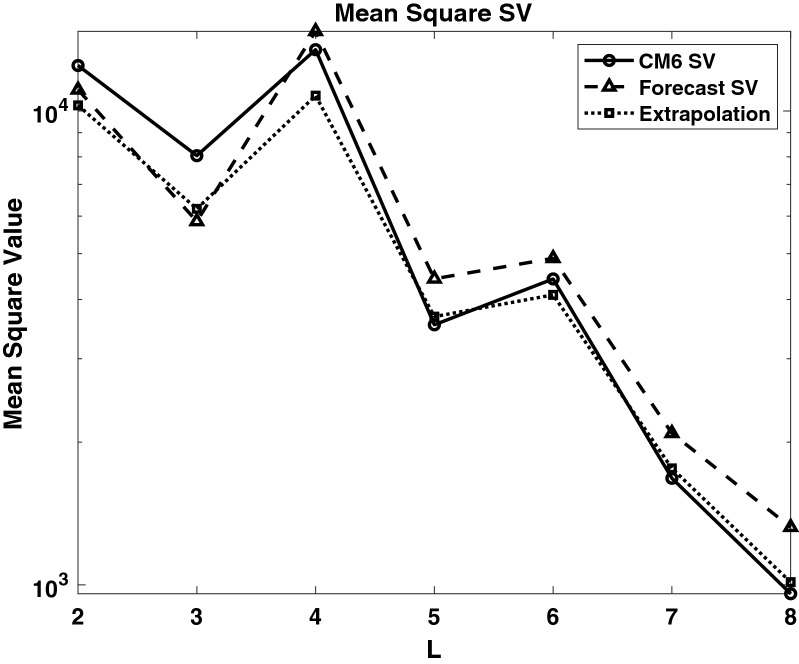

## 1. Introduction

It has long been observed that the Earth’s main magnetic field changes slowly in time (Bullard et al. 1950; Hide 1967; Bloxham and Gubbins 1985; Jackson et al. 2000; Lund 2018). This change, called the secular variation (SV), originates dominantly from the Earth’s iron-rich fluid outer core that is vigorously convecting, driven by thermo-chemical buoyancy released from Earth’s secular cooling and differentiation (geodynamo) (e.g. Lund and Olson 1987; Braginsky and Roberts, 1996). Over the past 25 years, great progress has been made in numerically modeling the geodynamo (Glatzmaier and Roberts 1995; Kagyama and Sato, 1997; Kuang and Bloxham 1997; Christensen et al. 2001; Jones et al. 2011; Matsui et al. 2016). These models can reproduce much of the qualitative aspects of the Earth’s magnetic field (Christensen et al. 2010), including dipole dominance, westward drift (Aubert et al. 2013) and occasional reversals (Olson 2007). Although it remains computationally prohibitive to simulate the geodynamo in the parameter regimes appropriate for the Earth’s core, the asymptotic properties emerged from numerical solutions with wide range of dynamo parameter values open the door for quantitative applications of numerical models to the geomagnetic field (e.g. Christensen and Aubert 2006; Christensen et al. 2010; Aubert and Fournier 2017; Kuang et al. 2017).

One such application is to use a numerical model to predict geomagnetic SV on time scales of several years and longer, if proper initialization is made using data assimilation techniques, as is done in numerical weather prediction (NWP). Forecast results from this geomagnetic data assimilation approach have already been used as predictive field candidates for previous IGRF models (Kuang et al. 2010; Fournier et al. 2015).

Obviously, this approach is different from other SV forecasts made without utilization of dynamo models. For example, Olsen, et al. (2010) modeled the time dependence of the Gauss coefficients with order 6 B-splines so that the predictive SV for 2010-2015 can be extrapolated from the coefficients derived from observations prior to 2010. While utilization of geodynamo models can take advantage of adding dynamically consistent motional induction in the outer core to forecast the SV, it also carries additional complications, such as model bias that results from the mismatch between the parameter values used in the models and those appropriate for the Earth’s core, and propagation of observational errors in both time and space. These difficulties have been analyzed through assimilation experiments with simplified systems (Sun et al. 2007; Fournier et al 2007) and observation system simulation experiments (OSSEs) (Liu et al 2007; Aubert and Fournier, 2011; Fournier, 2013). It is expected that improvements to both modeling and assimilation techniques will make geomagnetic forecast a growing contributor of the IGRF candidate models.

In this paper, we report our mean SV forecast for the period from 2020 to 2025. This effort differs from our earlier work for IGRF candidate predictive field model (Kuang et al. 2010) in two major areas: we assimilate Gauss coefficients from the CM6 geomagnetic field model (Sabaka et al in this special issue) for the period from 2000 to 2019; and the error covariance in our assimilation is determined through a large ensemble of assimilation solutions, and is updated in each analysis cycle. The two improvements can help produce more accurate SV forecasts and uncertainty estimates, since, for example, the utilization of a single field model can avoid any potential disagreement in different field models that may lead to inconsistent observation error estimates, and because the time varying error covariance can provide more consistent analysis for each cycle.

This paper is organized as follows: the algorithm of our forecasts is given in Section 2; testing and validation are presented in Section 3, followed by the forecasts and discussion.

## 2. Geodynamo model and data assimilation algorithm

The geomagnetic data assimilation system used for our SV forecasts is the NASA Geomagnetic Ensemble Modeling System (GEMS). It consists of a numerical geodynamo model (Kuang and Bloxham 1999; Kuang and Chao 2003), an ensemble Kalman filter (Evensen 2009; Sun and Kuang 2015). The model solves a set of nonlinear magnetohydrodynamic equations in a spherical shell domain. Details of the model are given in Kuang and Bloxham (1999), Kuang and Chao (2003) and Jiang and Kuang (2008). The core state in this model is defined with the fluid velocity field $\mathbf{v }$, the magnetic field $\mathbf{B }$ and the relative density anomaly $\Delta \rho$ that arises from temperature variations. These state variables are described by spherical harmonic expansions in the co-latitude $\theta$ and the longitude $\phi$, with the spherical harmonic coefficients given on discrete radial grid points. If we denote by $\mathbf{x }$ the vector of these coefficients, then the dynamo system can be symbolically represented as

1 $$\begin{aligned} \frac{\partial \mathbf{x }}{\partial t} = \mathbf{M }(\mathbf{x }) \end{aligned}$$

where $\mathbf{M }$ includes all linear and nonlinear model operators. GEMS employs a sequential ensemble Kalman filter (EnKF), described below, which is the result of many years of development that started with a simple one dimensional system (Sun et al. 2007) and an optimal interpolation (OI) system (Kuang et al. 2008, 2009). These systems have shown how geomagnetic data assimilation can be used to estimate optimal geodynamo model parameters and geomagnetic field model uncertainty (Kuang et al. 2010; Tangborn and Kuang 2015, 2018). This was done by running assimilation experiments with various parameter values and uncertainty estimates, and comparing the resulting forecasts with geomagnetic field models. In the present work, the model uses free-slip boundary conditions for the velocity field at the inner core boundary (ICB) and the core mantle boundary (CMB), and fixed-flux boundary conditions for temperature at the ICB and the CMB. In our geodynamo model, there is also a 20-km thick electrically conducting layer, called the $D''$-layer, at the base of the mantle, with a conductivity an order of magnitude lower that of the outer core. The $D''$-layer is homogeneous in the present study. Since the magnetic field is no-longer a potential field in this layer, the SV in the layer differs from those of the potential field (Greff-Lefftz and Legros 1995; Kuang et al. 2017). In our model, the Rayleigh number is $Ra = 1811$ and the Rossby number is $Ro = 1.25 \times 10^{-6}$. A single magnetic Prandtl number $Pr = 1$ is used throughout.

The observations used for GEMS are the Gauss coefficients $\{g_l^m, h_l^m\}$ (where *l* and *m* are spherical harmonic degrees and orders, and $m \le l$) from geomagnetic field models. They represent only the internally generated magnetic field. In the outer core and the $D''$-layer, the magnetic field can be decomposed into the toroidal ($\mathbf{B }_T$) and the poloidal ($\mathbf{B }_P$) components:

2 $$\begin{aligned} \mathbf{B } = \nabla \times (T_B {\hat{\mathbf{r }}}) + \nabla \times \nabla \times (P_B {\hat{\mathbf{r }}}) = \mathbf{B }_T + \mathbf{B }_P \end{aligned}$$

where $\hat{\mathbf{r }}$ is the radial unit vector, and $T_B$, $P_B$ are the toroidal and poloidal scalars and are described in GEMS as

3 $$\begin{aligned} \left[ \begin{array}{c} P_b \\ T_b \end{array} \right] \ =\ \sum _{l=1}^{L}\sum _{m=0}^{l}\, \left[ \begin{array}{c} b_l^m(r,t)\\ j_l^m(r,t) \end{array} \right] \,Y_l^m(\theta ,\phi )\ +\ C.C.\,, \end{aligned}$$

where $\{Y_l^m\}$ are the orthonormal spherical harmonic functions, and *C*.*C*. denotes the complex conjugate part of the expansion (similar expansions are also made for $\mathbf{v }$ and $\Delta \rho$). At the top of the $D''$-layer, i.e. at $r=r_d=3520 km$, the toroidal field vanishes, i.e. $j_l^m = 0$; but the poloidal field coefficients $b_l^m$ can be matched with the observations

4 $$\begin{aligned}b_l^m(r_d) = (-1)^m\frac{r_s^2}{l} \sqrt{\frac{2\pi }{2l+1}}\left( \frac{r_s}{r_d}\right) ^l \left( g_l^m - i h_l^m\right) \nonumber \\\quad \text{ for }\quad m\le l \le L_{obs}, \end{aligned}$$

where $r_s$ is the mean Earth surface radius, and $L_{obs}$ is the highest degree of the observed geomagnetic field.

In the rest of this paper, we denote the poloidal spectral coefficients in (4) that are derived from the field models by $(b_l^m)^o$ and the forecast $(b_l^m)^f$. The observations $\mathbf{y }^o = \left[ (b_l^m)^o \right]$ are assimilated into the forecast $\mathbf{x }^f$ at analysis time $t_a$ to produce the analysis $\mathbf{x }^a$:

5 $$\begin{aligned} \mathbf{x }^a(t_a) = \mathbf{x }^f(t_a) + \mathbf{K }\left[ \mathbf{y }^o - \mathbf{H }\cdot \mathbf{x }^f(t_a)\right] \end{aligned}$$

where $\mathbf{H }$ is the observation operator matrix and $\mathbf{K }$ is the gain matrix, defined by:

6 $$\begin{aligned} \mathbf{K } = \mathbf{P }^f\mathbf{H }^T \left[ \mathbf{HP} ^f \mathbf{H }^T + \mathbf{R }\right] ^{-1} \end{aligned}$$

where $\mathbf{P }^f$ is the forecast error covariance and $\mathbf{R }$ is the observation error covariance. The analysis $\mathbf{x }^a$ is then used as the initial state of the dynamo system (1) for the next forecast. The forecast error covariance is computed from an ensemble of forecasts with $N_{ens}$ members (Sun and Kuang 2015)

7 $$\begin{aligned} \mathbf{P }^f = \langle (\mathbf{x }^f - {\mu }_\mathbf{x }^f)(\mathbf{x }^f - {\mu }_\mathbf{x }^f)^T) \rangle \end{aligned}$$

where ${\mu }_\mathbf{x }^f$ is the ensemble mean forecast state. In our forecasts here, we take only the diagonal part of the observation error covariance $\mathbf{R }$ and is defined simply as the estimated variance of the field models, $((\sigma _l^m)^o)^2$. The time dependent model for the observation error standard deviation is

8 $$\begin{aligned} (\sigma _l^m)^o = \alpha ||b_l^m ||(l)(l+1)/2 \end{aligned}$$

where $\alpha = 0.1$ before 1900, $0.001 \le \alpha \le 0.1$ with an exponential decrease from 1900 to 2000, and where $\alpha = 0.001$ afterwards. This observation error model accounts for the increased accuracy of geomagnetic models at later times, as well as the higher relative errors for the higher degrees. It has been tested through a number of numerical experiments with the goal of minimizing forecast errors, and is therefore regarded as an appropriate estimate relative to our geodynamo forecasts.

The geomagnetic field models that are used to provide the Gauss coefficients for our assimilation, and their maximum degree assimilated ($L_{obs}$) are shown in Table 1.

**Table 1:** Geomagnetic field models and the highest degrees used in assimilation

Observations	Period	$L_{obs}$
*gufm1*:	1590–1960	4
CM4:	1960-2000	8
CM6:	2000-2019	13

Details of *gufm1*, CM4 and CM6 can be found in Jackson et al. (2000), Sabaka et al. (2004), and Sabaka et al. (2020) respectively. We only summarize here that the latter model uses a “comprehensive inversion” technique which co-estimates parameters for the Earth’s internal field, using estimates of systematic and random errors, so as to produce an optimal separation of the internal and external fields. The values of *L* *obs* for each model here decrease further back in time, so as to avoid introducing errors from the smoothing or regularization in the earlier models. These values may in fact not be optimal, but our previous experimentation (Tangborn and Kuang 2018) has shown that reducing them for the earlier field models can lead to more accurate forecasts in the modern era.

The ensemble is initialized in 1590 using a long free model run from which uncorrelated ensemble members are generated, and subsequent analyses are computed every 20 years until 2000, when the analysis cycle is reduced to one year (the start of the forecast period using the CM6 model). $\mathbf{P }^f$ is updated with the forecasts at each analysis time $t_a$, but $\mathbf{H }$ is only updated if different $L_{obs}$ is chosen for making the analysis. We use an ensemble of 400 for these experiments, and we have found this size to result in forecast accuracy only slightly better than smaller ensembles of 200 or 256, and very similar to an ensemble of 512. Any further increases in the ensemble size would result in larger computational costs that outweigh small increases in accuracy.

Geomagnetic forecasts using a geodynamo model will naturally contain significant forecast errors due to mismatches in the dynamo parameter values and numerical approximations, and these can grow significantly during a multi-year forecast. But because these error sources develop on relatively long timescales, we can assume that the model error itself varies on time scales much longer than, e.g. the 5-year forecast period. This assumption allows us to implement a bias correction scheme for the SV prediction by producing a set of staggered forecasts, and then take differences between them to reduce substantially the bias (Kuang et al. 2010). The forecast *y*, of the observed poloidal field is defined as the projection of the state vector, $\mathbf{x }^f$ onto the observation space:

9 $$\begin{aligned} \mathbf{y }^f = \mathbf{Hx} ^f \end{aligned}$$

We define the forecast error, ${\epsilon }(t)$, as the difference between the forecast state, $\mathbf{y }^f(t)$, and the true state, $\mathbf{y }^t(t)$:

10 $$\begin{aligned} \mathbf{y }^f(t) = \mathbf{y }^t(t) + {\epsilon }(t). \end{aligned}$$

The forecast error is a combination of the model error and the growth of initial state errors, but we assume that the model errors remain the largest component. Consider a forecast that is initialized from an analysis at time $t_a$ and produces a forecast at time $t_a + \delta t$. We estimate the SV from the change in the geomagnetic field after time $\delta t$:

11 $$\begin{aligned} \mathbf{y }^f(t_a + \delta t) - \mathbf{y }^a(t_a) = \mathbf{y }^t(t_a + \delta t) - \mathbf{y }^t(t_a) + {\epsilon }(t_a + \delta t) - {\epsilon }(t_a) \approx ({\dot{\mathbf{y }}} + {{\dot{\epsilon }}}) \delta t \end{aligned}$$

where ${\dot{\mathbf{y }}}$ is the SV and ${{\dot{\epsilon }}}$ is the SV error growth rate. We can reduce this error term by making use of the assumption of slowly varying model error and considering staggered forecasts that begin at two slightly different analysis times $t_a$ and ${\tilde{t}}_a$, where $| t_a - {\tilde{t}}_a | < \delta$. This gives us forecasts at times $t_a + \delta t$ and ${\tilde{t}}_a + \delta t$ in terms of the true state:

12 $$\begin{aligned} \begin{array}{l} \mathbf{y }^f(t_a + \delta t) = \mathbf{y }^t (t_a + \delta t) + {\epsilon }(t_a + \delta t) \\ \mathbf{y }^f({\tilde{t}}_a + \delta t) = \mathbf{y }^t ({\tilde{t}}_a + \delta t) + {\epsilon }({\tilde{t}}_a + \delta t) \end{array} \end{aligned}$$

The difference between these two sequences was shown to approximately remove the forecast error (Kuang et al. 2010), if the change in the model error between $t_a + \delta t$ and ${\tilde{t}}_a + \delta t$ is small compared to the SV. So we can calibrate the SV forecast with the following scheme

13 $$\begin{aligned} \tilde{{\dot{\mathbf{y }}}}^f (t) = \frac{1}{\tau }_a \left[ \mathbf{Hx} ^f (t_a + \delta t) - \mathbf{Hx} ^f ({\tilde{t}}_a + \delta t)\right] \end{aligned}$$

where $\tau _a = |t_a - {\tilde{t}}_a| = 1 year$, and $t = t_a + \delta t$ is the time of the SV forecast. We take $\delta t = 5$ years for these forecasts.

The details of the bias correction scheme can be found in Kuang et al. (2010).

## 3. Validation Experiments

We have validated the methodology by computing SV forecasts for the period 2009-2015. Two forecast experiments were conducted using the same assimilation run that started in 1590, and from 2000 it is carried forward with one year forecast and analysis cycles. In the first experiment (A), the assimilation is computed from 2000 to 2008; the analysis in 2008 is then used to make forecasts for the period 2009-2014. The second experiment (B) is similar, but the assimilation is computed from 2000 to 2009; and the analysis in 2009 is used to make the forecast for the period 2010-2015. The final SV forecasts are then performed with these two staggered forecasts:

14 $$\begin{aligned} \tilde{{\dot{\mathbf{y }}}}_{AB}(t_f) = \left[ \mathbf{y }_2(t_f+\tau _a) - \mathbf{y }_1(t_f)\right] /\tau _a \end{aligned}$$

The SV for the Gauss coefficients ($g_l^m,h_l^m$) are then computed as follows. We show the details for $g_l^m$, while $h_l^m$ follow the same procedure. In our assimilation, we utilize the scaled coefficients

15 $$\begin{aligned} G_l^m = g_l^m/g_1^0 \end{aligned}$$

for the SV forecasts. This is done because the parameters in the geodynamo model are far from those in the Earth’s core, and assimilating $g_1^0$ would force the model into a regime that the current model grid cannot resolve. We first compute $G_l^m$ from our forecasts, and their time variation ${\dot{G}}_l^m$ via (14). The SV Gauss coefficients can then be calculated via

16 $$\begin{aligned} {\dot{g}}_l^m = \frac{d}{dt} (g_1^0 G_l^m) = g_1^0{\dot{G}}_l^m + {\dot{g}}_1^0 G_l^m. \end{aligned}$$

We then approximate the axial dipole coefficient $g_1^0$ and its time derivative ${\dot{g}}_1^0$ in (16) with the values from CM6 (Sabaka et al. 2020) as follows:

17 $$\begin{aligned}&{\dot{g}}_1^0 = \left[ (g_1^0)^{CM6}(2010) - (g_1^0)^{CM6} (2009)\right] /(1 year), \nonumber \\&\quad g_1^0(2009+t) = (g_1^0)^{CM6}(2009) + t {\dot{g}}_1^0. \end{aligned}$$

**Fig. 1:**
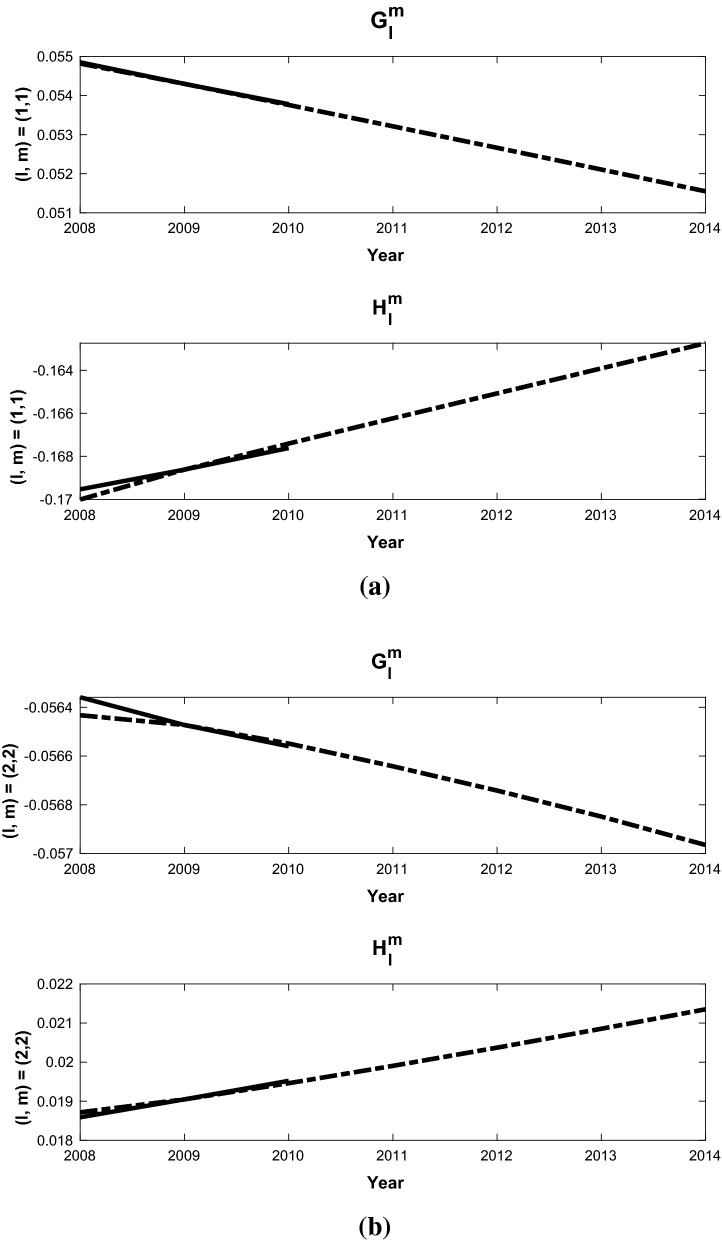
SV validation forecasts (dashed line) and CM6 values (solid line) for 2010-2015 for scaled Gauss coefficients $l=1, m=1$ (**a**) and $l=2, m=2$ (**b**) ($G_l^m$, $H_l^m$) normalized using $g_1^0$ from 2009. The resulting SV values are shown in Table 2

**Fig. 2:**
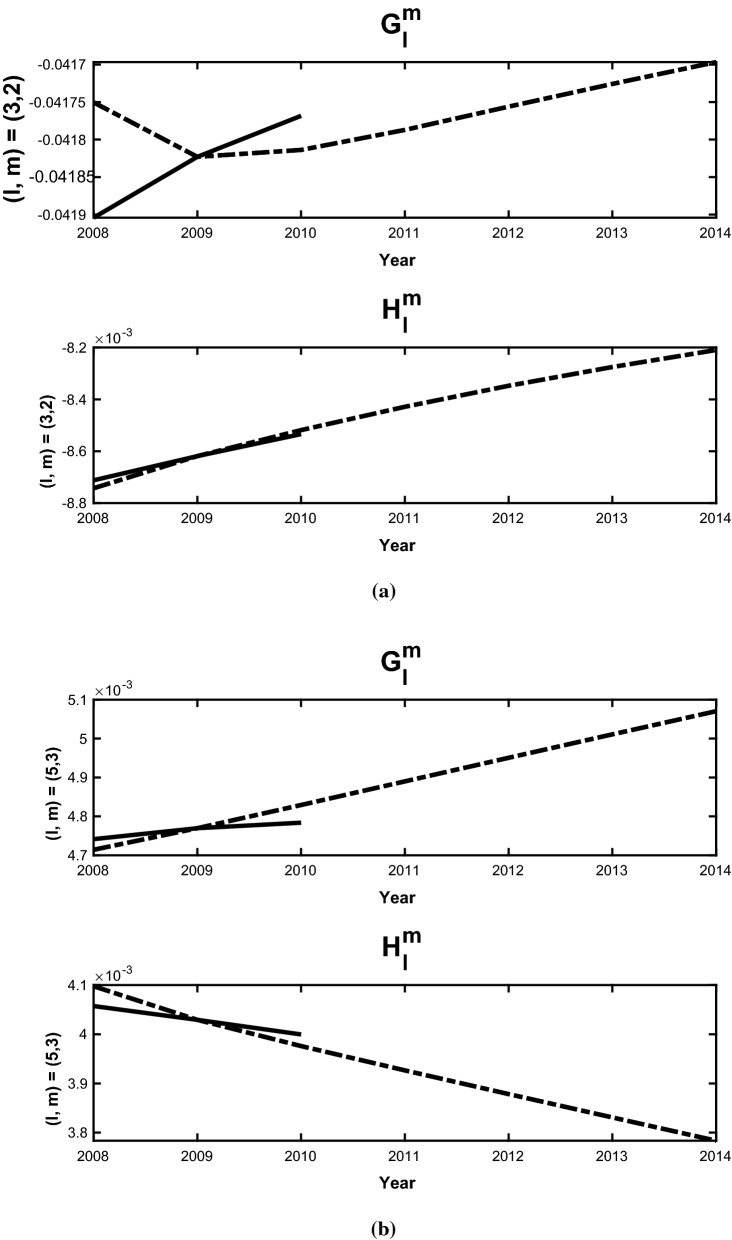
Same as Fig. 1 but for $l=3, m=2$ and $l=5, m=3$

The formulations (13)–(17) are the basis for the forecasts of the mean SV for the period 2010-2015, and are compared with CM6 for each degree *l* and order *m* up to $l=8$. In this case the years in A correspond to ${\tilde{\tau }}$ and those in B to $\tau$. Some examples are shown in Figs. 1, which demonstrate the forecast field, $\tilde{{\dot{\mathbf{y }}}}^f$, for different *l*, *m*. The slope of each dashed line is the scaled forecast from (15) while the solid line is the scaled SV computed from CM6 coefficients. The coefficients shown here are representative of the range in accuracy obtained by the ensemble forecast and bias correction algorithm. The $(l=m=1)$ and $(l=m=2)$ coefficients in Fig. 1 are typical of coefficients that predict the CM6 field model with a higher degree of accuracy than others. The $(l=3, m=2)$ in Fig. 2(a) is much less accurate accurate case (for $g_l^m$) with a slope of opposite sign, while $(l=5, m=3)$ has a typical mid-range accuracy for the dimensionless scaled SV forecasts. The final dimenional SV values determined by the validation calculations are shown in Table 2 These bias corrected SV forecasts are computed each year out to 2015, and the five year time average SV can be computed from them.

**Fig. 3:**
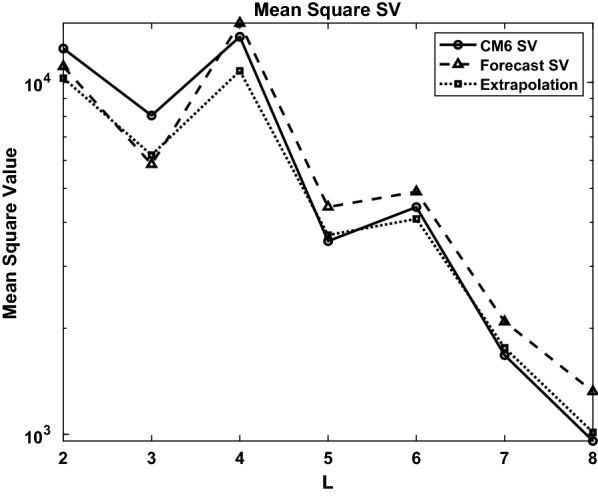
Mean square SV 2009-2015 vs. harmonic degree for forecast (dashed line), CM6 (solid) and CM6 extrapolation from 2009-2010 (dotted)

We summarize the SV forecasts by plotting mean square SV fields ($MS_{SV}$), shown in Fig. 3 for the EnKf forecast (dashed line), CM6 (solid line) and an extrapolation of CM6 from 2009 (dotted line) as a function of degree *L*. The mean square SV is computed using

18 $$\begin{aligned} MS_{SV} = \sum _{m=0}^l \frac{(l+1)^2}{(2l+1)}\left[ ({\dot{g}}_l^m)^2 + ({\dot{h}}_l^m)^2\right] \end{aligned}$$

Here it can be seen that the EnKF forecasts are more accurate than the extrapolated SV for $L=2$ and shows similar accuracy for $L=3,4$, while the accuracy for $L=5$ to $L=8$ is significantly worse. The individual coefficients for these three cases are given in Table 2. We discuss the possible sources of error and potential solutions in the Summary and Conclusions section.

**Table 2:** SV for 2010-2015 validation experiments from CM6, the GEMS EnKF, and extrapolations of CM6 from 2009-2010

L	m	CM6		Extrapolation		Forecast	
1	1	16.9980	− 29.6062	16.8822	−28.9857	16.87	−36.28
2	0	−10.0399	0.0000	−11.5499	0.0000	−4.39	0.00
2	1	−2.7965	−27.4081	−3.9213	−22.8400	−2.18	−26.57
2	2	1.6279	−13.2957	2.7249	−13.3551	2.43	−13.71
3	0	2.2055	0.0000	1.1431	0.0000	3.86	0.00
3	1	−5.1435	9.0073	−3.9088	8.7466	0.78	8.68
3	2	−1.2684	−1.3568	−2.8669	−2.8496	−1.33	−2.37
3	3	−10.3680	−0.3712	−7.7075	−1.9579	−7.70	−2.22
4	0	−1.0547	0.0000	−1.3856	0.0000	2.95	0.00
4	1	0.9199	−0.5882	1.9925	0.2234	1.41	1.34
4	2	−9.2351	4.5314	−8.9866	3.0664	−10.48	3.53
4	3	4.3963	3.2985	4.4430	3.7606	5.91	2.64
4	4	−3.8282	−3.9203	−2.0291	−0.7498	−2.00	−1.84
5	0	−0.3896	0.0000	−0.5386	0.0000	1.21	0.00
5	1	0.5725	0.4570	0.5931	0.4944	1.48	0.29
5	2	−1.5870	1.6027	−1.5356	1.4798	−0.63	1.01
5	3	0.0359	−0.2325	−0.7754	0.8731	−1.72	1.46
5	4	1.1619	3.2020	1.2403	3.7308	−0.90	3.93
5	5	2.4740	−0.1790	1.3844	−0.6087	1.73	0.04
6	0	−0.6603	0.0000	−0.2814	0.0000	0.19	0.00
6	1	−0.2173	0.0588	−0.2364	−0.0351	−0.55	0.00
6	2	−0.6162	−2.1738	−0.3307	−2.2186	−0.73	−2.39
6	3	2.3154	−0.5691	2.0345	−0.4257	1.62	−0.52
6	4	−1.2210	−0.0776	−1.5997	−0.5149	−1.91	−0.12
6	5	0.0105	0.8680	−0.1500	0.6775	−0.32	0.39
6	6	1.4449	1.3886	1.7602	0.5885	2.10	1.26
7	0	0.1644	0.0000	0.1883	0.0000	0.70	0.00
7	1	−0.1965	0.7082	−0.1043	0.5869	0.28	0.12
7	2	−0.4427	0.3374	−0.5881	0.2778	−0.74	0.47
7	3	1.3211	−0.1861	1.3548	−0.1108	0.72	−0.13
7	4	0.2199	−0.1032	0.2192	−0.1536	−0.44	−0.24
7	5	−0.2284	−0.7514	0.0913	−0.7550	0.27	−0.91
7	6	−0.9005	0.0212	−0.8291	−0.3096	−1.34	−0.34
7	7	0.3188	0.1894	0.5567	0.2978	0.61	0.08
8	0	−0.0753	0.0000	−0.1036	0.0000	−0.12	0.00
8	1	0.1342	−0.1632	0.1065	−0.0524	0.07	0.17
8	2	−0.4481	0.3643	−0.5856	0.1758	−0.64	0.00
8	3	0.4864	0.2678	0.3043	0.4529	0.58	0.51
8	4	−0.2490	0.5588	−0.2602	0.4869	−0.08	0.33
8	5	0.3437	−0.1072	0.2841	0.0695	0.45	0.12
8	6	0.1812	−0.2571	0.2611	−0.1151	0.20	−0.15
8	7	−0.3853	0.3267	−0.5167	0.3988	−0.47	0.64
8	8	0.2976	0.1551	0.2287	0.3353	0.33	0.33

## 4. Mean SV for 2020–2025

We repeat the same method to make the SV forecast for the period from 2020 to 2025, except that we use three staggered forecasts, again using an ensemble of 400 members each. Starting from the analysis computed for 2000, we have continued the assimilation and forecast (with the annual analysis cycle) forward in time for three sets of experiments, with the final analysis times in 2017, 2018 and 2019, and the forecasts are performed in the next six years. The experiments, labeled A, B and C; are summarized in Table 3.

**Table 3:** Staggered forecast experiments

	$t_a$	$t_{f_1}$	$t_{f_2}$	$t_{f_3}$	$t_{f_4}$	$t_{f_5}$	$t_{f_6}$
Exp.A	2017	2018	2019	2020	2021	2022	2023
Exp.B	2018	2019	2020	2021	2022	2023	2024
Exp.C	2019	2020	2021	2022	2023	2024	2025

With these three sets of forecasts, we can use up to the following three different combinations of the differences between two staggered forecasts:

19 $$\begin{aligned} \begin{array}{l} \tilde{{\dot{\mathbf{y }}}}_{BA} = (\mathbf{y }_B - \mathbf{y }_A)/\tau _a, \\ \tilde{{\dot{\mathbf{y }}}}_{CB} = (\mathbf{y }_C - \mathbf{y }_B)/\tau _a, \\ \tilde{{\dot{\mathbf{y }}}}_{CA} = (\mathbf{y }_C - \mathbf{y }_A)/(2\tau _a), \end{array} \end{aligned}$$

where again $\tau _a = 1$ year and the AC experiments are 2 years apart. The SV for the forecast Gauss coefficients is computed as before, along with the SV for CM6.

**Fig. 4:**
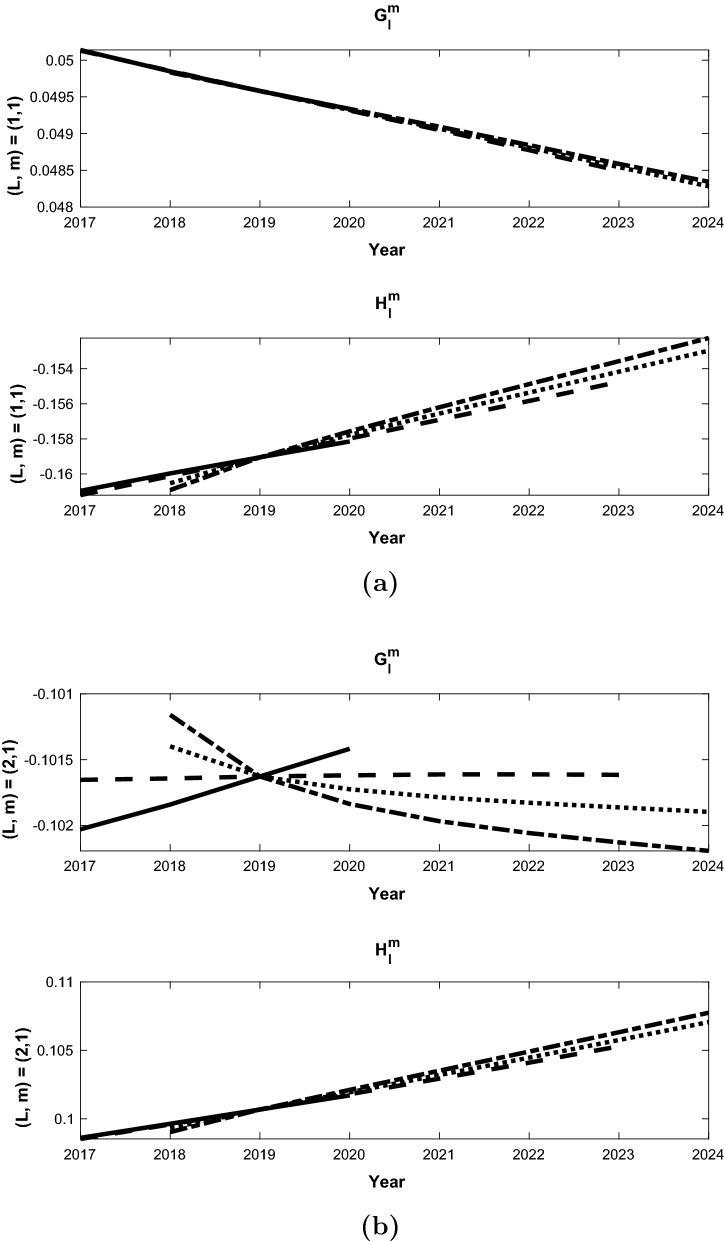
Bias corrected forecasts of Gauss coefficients from differences between forecast sequences that start in 2016, 2017 and 2018. Each bias corrected forecast starts in the year of the later sequence. The solid line shows the 2020 forecast from the Goddard candidate model for IGRF-2020-SV, while the dashed (experiment A - experiment B), dash-dot (B-C) and dotted (A-C) lines show the bias corrected forecasts from our assimilation that use differences between the different forecast series. Panel **a** shows the coefficients for $l=1$ and $m=1$, while panel **b** shows the coefficients for $l=2$, $m=1$. Each panel shows the the bias corrected forecasts for $G_l^m$ (upper) and $H_l^m$ (lower) from Eq. 19

**Fig. 5:**
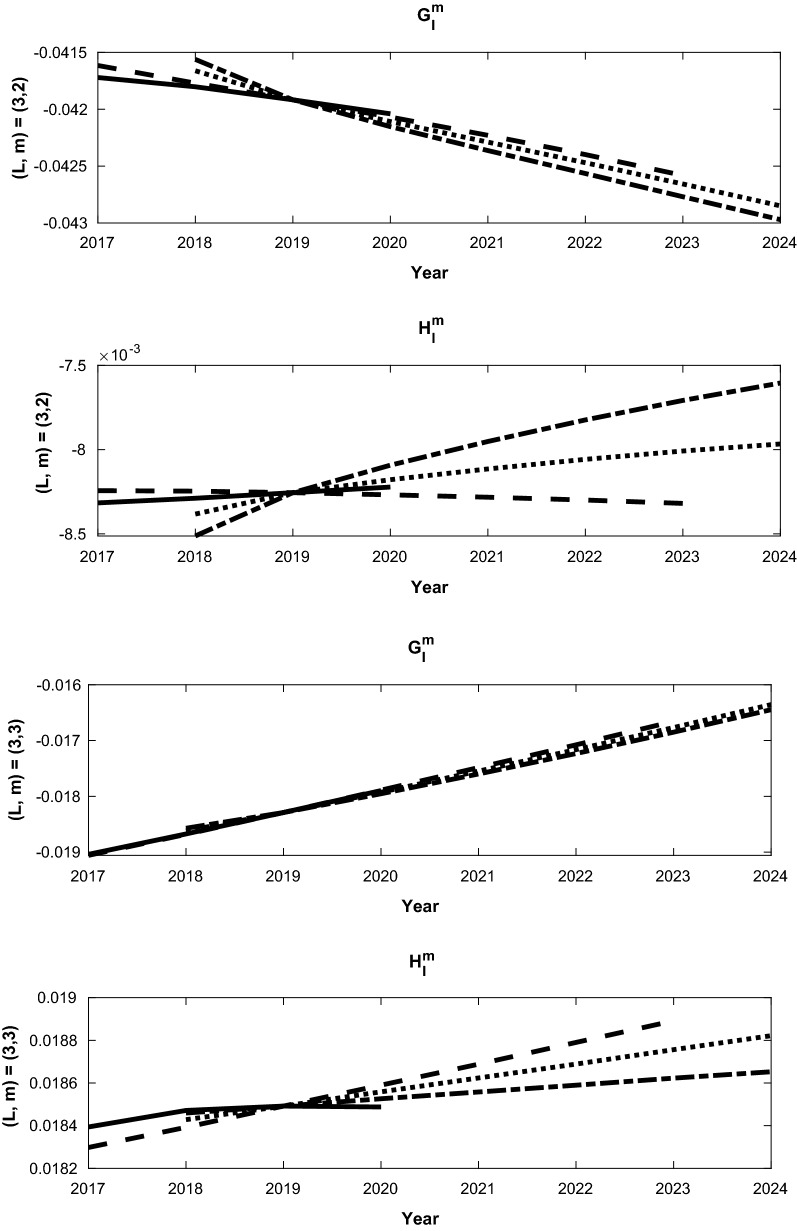
Same as Fig. 4, except panel (**a**) shows the coefficients for $l=3$ and $m=2$, while panel (**b**) shows the coefficients for $l=3$ and $m=3$

**Fig. 6:**
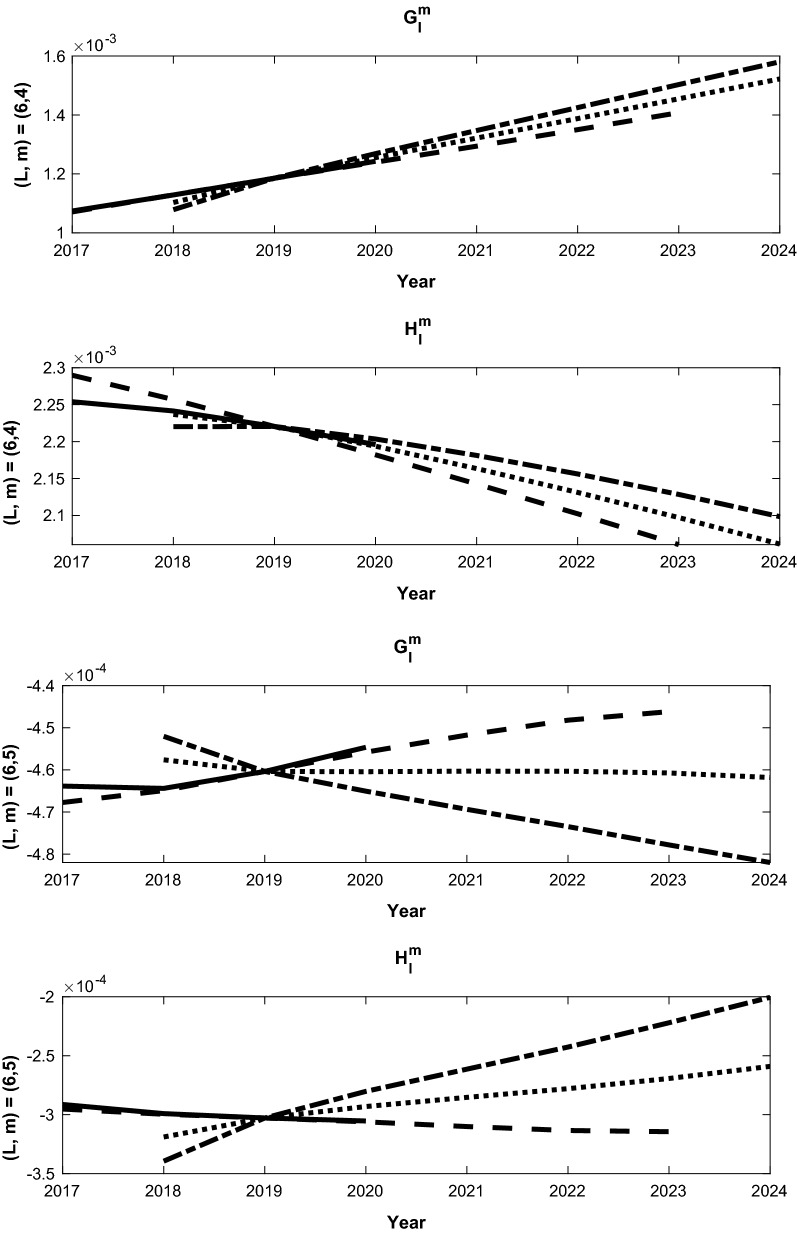
Same as Fig. 4, except panel (**a**) shows the coefficients for $l=6$ and $m=4$, while panel (**b**) shows the coefficients for $l=6$, $m=5$

The bias corrected SV forecast for 2025 is a combination of the different forecasts, BA, CB or CA from (19). There are two main reasons for our last analysis ending at 2019: one is that the predictive field from CM6 for 2020 will be used as the initial condition for our forecast calibration; the other is the SV from CM6 for the period 2019-2020

20 $$\begin{aligned} (\dot{g_l^m})^{CM6} = \left[ (g_l^m)^{CM6}(2020) - (g_l^m)^{CM6} (2019) \right] /(1 year) \end{aligned}$$

can be used to estimate our model bias. Some representative forecasts that demonstrate the bias correction approach and comparison to the CM6 2020 forecast are shown in Figs. 4, 5, 6. Each figure consists of the three uncorrected forecasts for $g_l^m$ and $h_l^m$, along with the 2017-2020 forecast from CM6, on top (A,B and C) and the bias corrected forecasts below (BA, CB and CA). The corrected forecasts in each second set in each figure are combinations of the uncorrected forecasts, as described in (19). The slope of each line is the time varying SV, and we confirm the consistancy of each of the experiments with the CM6 SV in each case. For example, Fig. 4(a) shows the forecasts for $(l,m) = (1,1)$, and the SV for $g_1^1$ requires a negligble correction since the three experiments have essentially the same SV. The SV for $h_1^1$ shows a small change from the bias correction and the *AB* correction is used since its slope is closest to the CM6 estimate. The other two SV estimates are then discarded.

The final calibrated mean SV for the period 2020–2025 is computed as the following: first, the optimal annual SV forecasts ${\dot{g}}_l^{m(f)}$ are made from our data assimilation system for 2020-2024, with the same procedure discussed in the previous section. Then, the time varying Gauss coefficients are computed via

21 $$\begin{aligned} g_l^{m(f)}(2020+k)= & {} (g_l^m)^{CM6}(2020) \nonumber \\&+ \sum _{l=0,k-1} {\dot{g}}_l^{m(f)}(2020+k) \times (\tau _a), \quad \text{ for }\quad k = 1,2,\cdot ,5. \end{aligned}$$

Finally, the mean annual SV Gauss coefficients are determined via

22 $$\begin{aligned} \overline{{\dot{g}}_l^{m(f)}} = \left[ g_l^{m(f)}(2025)-g_l^{m(f)}(2020)\right] /5. \end{aligned}$$

The uncertainties are determined by the standard deviation of the forecast ensemble. The final results are given in Table 4 along with the associated uncertainties, which are the ensemble standard deviations from the ensemble forecast.

**Table 4:** Table of the mean SV Gauss coefficients for $g_l^m,h_l^m$ (with the unit nT/year). The axial dipole SV ${\dot{g}}_1^0$ is given by Sabaka et al. (2020)

l	m	${\dot{g}}$	${\dot{h}}$	${\dot{g}}$ uncert	${\dot{h}}$ uncert
1	0	4.78	0.00	0.0056	0.0000
1	1	8.18	−32.26	0.0053	0.0084
2	0	−5.84	0.00	0.0054	0.0000
2	1	−0.50	−33.51	0.0073	0.0076
2	2	−3.87	−18.41	0.0042	0.0063
3	0	6.49	0.00	0.0057	0.0000
3	1	3.41	6.05	0.0038	0.0029
3	2	4.72	0.44	0.0042	0.0040
3	3	−10.90	−0.87	0.0034	0.0033
4	0	−1.17	0.00	0.0023	0.0000
4	1	−1.31	0.30	0.0029	0.0033
4	2	−6.34	6.82	0.0016	0.0022
4	3	4.91	4.66	0.0031	0.0030
4	4	−4.07	−5.10	0.0019	0.0018
5	0	−0.31	0.00	0.0028	0.0000
5	1	−0.17	0.42	0.0014	0.0013
5	2	−0.78	3.19	0.0016	0.0019
5	3	−0.08	0.46	0.0013	0.0012
5	4	2.57	3.20	0.0020	0.0012
5	5	1.24	−0.30	0.0010	0.0011
6	0	−0.60	0.00	0.0007	0.0000
6	1	−0.22	0.18	0.0010	0.0010
6	2	−0.94	−1.70	0.0008	0.0007
6	3	2.22	−0.65	0.0011	0.0010
6	4	−1.63	0.95	0.0008	0.0007
6	5	−0.11	0.08	0.0007	0.0006
6	6	0.87	0.82	0.0007	0.0007
7	0	−0.09	0.00	0.0006	0.0000
7	1	0.20	0.45	0.0005	0.0004
7	2	−0.28	0.49	0.0006	0.0005
7	3	0.91	−0.81	0.0004	0.0004
7	4	−0.55	0.05	0.0008	0.0006
7	5	−0.61	−1.01	0.0003	0.0003
7	6	−0.84	0.25	0.0004	0.0005
7	7	0.83	0.33	0.0004	0.0003
8	0	0.05	0.00	0.0003	0.0000
8	1	0.15	−0.36	0.0003	0.0003
8	2	−0.30	0.47	0.0003	0.0002
8	3	0.57	−0.02	0.0003	0.0004
8	4	−0.21	0.64	0.0003	0.0003
8	5	0.52	0.19	0.0003	0.0003
8	6	0.09	−0.32	0.0002	0.0002
8	7	−0.46	0.56	0.0003	0.0003
8	8	0.38	−0.08	0.0002	0.0002

## 5. Summary and Conclusions

We have used an EnKF to forecast the geomagnetic secular variation (Table 4) for the time period 2020–2025, using 400 ensemble members. The development of ensemble forecasting techniques has contributed to the SV forecasts, particularly due to the ability to estimate uncertainty, which is newly available with this geomagnetic forecast. The validation forecast for 2015 shows that the SV forecasts compare favorably to the SV from CM6 (relative to an extrapolated SV from 2009-10) for degrees 2-4 (degree 1 is excluded because the forecasts are scaled by $(g_1^0)^o$), but show poorer accuracy for degrees 5-8. The lower accuracy in the higher degree terms is likely due to shorter timescales for model bias for these coefficients, which could violate the assumption that the model bias is constant over a period of several years. This would make it difficult for the model to predict geomagnetic jerks (Aubert and Finlay 2019). The uncertainty estimates provided with the candidate model are seen to be too low, based on the validation experiment, in part due to limitations on the constant bias assumption. However, the 2020-25 SV used a series of 3 forecasts which helped eliminate outlier forecasts, which benefited the final SV forecast, whereas the 2010-2015 forecast only used 2 forecasts. While this is the first attempt at an SV forecast using this EnKF system, we expect that improvements can be made to the forecast in the future. In particular, we plan to gradually increase the range of geodynamo parameter values, and explore alternate timescale relationships between the dynamo model and the geomagnetic field models.

The large ensemble has placed a constraint on this forecast, because at present it is computationally challenging to run the ensemble assimilation system for 7000 years, as was done in Kuang et al. (2010) using an OI assimilation algorithm. Future work will investigate the use of localization schemes that would enable the use of a much smaller ensemble that could be run for longer time periods.

## Data Availability

The data (IGRF candidate Gauss coefficients) is included as a table in this paper. All of the data presented in this paper is already publicly available (for example, the field models), or will be made available upon publication (the SV Gauss coefficients contained in this paper).

## Abbreviations

CM4: Comprehensive Model version 4
CM6: Comprehensive Model version 6
EnKF: Ensemble Kalman Filter
GEMS: Geomagnetic Ensemble Modeling System
IGRF: International Geomagnetic Reference Field
NWP: Numerical Weather Prediction
OSSE: Observing System Simulation Experiment
SV: Secular variation
